# Association between atrial fibrillation, atrial enlargement, and left ventricular geometric remodeling

**DOI:** 10.1038/s41598-018-24875-1

**Published:** 2018-04-23

**Authors:** Yuta Seko, Takao Kato, Tetsuya Haruna, Toshiaki Izumi, Shoichi Miyamoto, Eisaku Nakane, Moriaki Inoko

**Affiliations:** 1 Cardiovascular Center, The Tazuke Kofukai Medical Research Institute, Kitano Hospital, Osaka, Japan; 2 Department of Cardiovascular Medicine, Kyoto University Graduate School of Medicine, Kyoto, Japan

## Abstract

This study investigated the relationship between atrial fibrillation (AF) and left ventricular (LV) geometric patterns in a hospital-based population in Japan. We retrospectively analyzed 4444 patients who had undergone simultaneous scheduled transthoracic echocardiography (TTE) and electrocardiography during 2013. A total of 430 patients who had findings of previous myocardial infarctions (n = 419) and without the data on body surface area (n = 11) were excluded from the study. We calculated the LV mass index (LVMI) and relative wall (RWT) and categorized 4014 patients into four groups as follows: normal geometry (n = 3046); concentric remodeling (normal LVMI and high RWT, n = 437); concentric hypertrophy (high LVMI and high RWT, n = 149); and eccentric remodeling (high LVMI and normal RWT, n = 382). The mean left atrial volume indices (LAVI) were 22.5, 23.8, 33.3, and 37.0 mm/m^2^ in patients with normal geometry, concentric remodeling, concentric hypertrophy, and eccentric hypertrophy, respectively. The mean LV ejection fractions (LVEF) were 62.7, 62.6, 60.8, and 53.8%, respectively, whereas the prevalence of AF was 10.4%, 10.5%, 14.8%, and 16.8% in patients with normal geometry, concentric remodeling, concentric hypertrophy, and eccentric hypertrophy, respectively. In conclusion, the prevalence of AF was increasing according to LV geometric remodeling patterns in association with LA size and LVEF.

## Introduction

Left ventricular (LV) hypertrophy (LVH) may be considered a compensatory effect since increasing LV wall thickness reduces LV wall stress^1^. However, as LVH progresses, it is associated with considerable cardiovascular (CV) morbidity and mortality^2,3^. Recent studies have focused on the prognostic impact of more subtle LV geometric abnormalities. Patterns of LVH and geometric remodeling have previously been investigated extensively in patients with hypertension and valvular heart diseases^4,5^. Concentric LVH has a high mortality risk with preserved ejection fraction (EF)^6,7^ or a high mortality risk in patients without regression of abnormal LV geometry^8^; other studies have reported that relative wall thickness has less impact on prognosis in patients with coronary heart disease^3^.

LVH and LV dilatation cause an in increase in end-diastolic pressure, followed by enlargement of the left atrium^9^. Atrial fibrillation (AF) is more prevalent among older people with pronounced morbidity and mortality^10–13^. AF can cause heart failure, affect quality of life, lengthen the hospitalization period, and increase mortality^14^. Left atrial (LA) remodeling, LA enlargement, and LV remodeling are related to AF development^15^. However, the association between LV geometric patterns, LA enlargement, and AF remains unknown. Therefore, in this study, we investigated the association between LV geometric patterns, LA enlargement, and AF.

## Methods

### Study population

**Figure 1:**
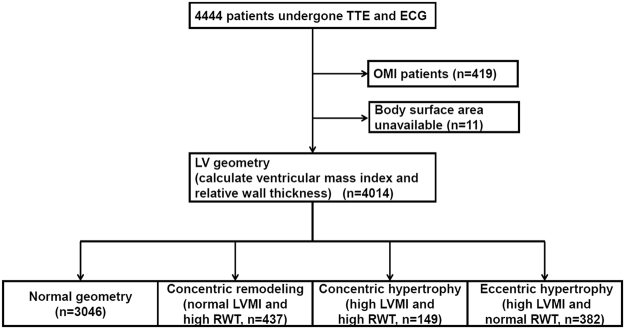
Flowchart of the study population. Abbreviations: TTE = transthoracic echocardiography; ECG = electrocardiography; LV = left ventricular; LVMI = LV mass index; RWT = relative wall thickness; and OMI = old myocardial infarction.

We retrospectively analyzed 4444 patients who had undergone simultaneous scheduled transthoracic echocardiography (TTE) and electrocardiography (ECG) at the Cardiovascular Center of Kitano Hospital during 2013. A flowchart of the study population is shown in Fig. 1. A total of 430 patients who had findings of previous myocardial infarctions (n = 419) and without the data on body surface area (n = 11) were excluded from the study, because the old myocardial infarction affected their wall thickness and other data from the TTE. On the basis of the data from the TTE and ECG examinations in addition to the catheter database, we identified patients who had a previous myocardial infarction. The ECG and TTE were ordered by each physician.

The research protocol was approved by the institutional review board of Kitano Hospital (approval no.: P16-02-005). Informed consent was not obtained from each patient since this was a retrospective study. The study protocol conforms to the ethical guidelines of the 1975 Declaration of Helsinki as reflected in a priori approval by the institution’s human research committee. Patients’ records and information were anonymized and de-identified before analysis.

### Data collection

From the TTE database, we extracted data for patients’ wall thickness, LV diastolic diameter (LVDd), E/e’, LA diameter (LAD), LA volume index (LAVI), and LV ejection fraction (LVEF). We also extracted the body surface area from the TTE report. From the ECG database, we extracted cardiac rhythm and recorded it as it was documented. Therefore, we could not determine whether the AF was paroxysmal or persistent.

The LV mass was calculated using the formula recommended by the American Society of Echocardiography (ASE), and it was indexed to the body surface area as follows: LV mass = 0.8 × 1.04 [(LVDd + LVPWTd + IVSTd)^3^ − (LVDd)^3^] + 0.6, where LVDd was the LV diastolic diameter, IVSTd was the diastolic interventricular septal wall thickness, and LVPWTd was the diastolic LV posterior wall thickness. In line with the ASE recommendations, a high LV mass index (LVMI) was defined as >115 g/m^2^ for male patients and >95 g/m^2^ for female patients. Relative wall thickness (RWT) was calculated using the following formula: (2 × LVPWTd)/(LVDd), which permits physicians to categorize an increase in the LV mass as either concentric (RWT >0.42) or eccentric (RWT ≤0.42) hypertrophy and identify concentric remodeling (a normal LV mass with an increased RWT)^16,17^. The LAVI was calculated using the biplane area-length method and body surface area and defined high as a value >42 mL/m^2^^14,15^. Data from two-dimensional TTE were analyzed at baseline. The LVEF was measured using the Teichholz method or the modified Simpson rule method and defined low as LVEF <50%.

We then categorized 4014 patients into four groups as follows (Fig. 1): normal geometry (n = 3046), concentric remodeling (normal LVMI and high RWT, n = 437), concentric hypertrophy (high LVMI and high RWT, n = 149), and eccentric hypertrophy (high LVMI and normal RWT, n = 382).

We extracted patients’ data from the electronic medical data at our institution, including age, sex, and type of disease, i.e., hypertension (International Statistical Classification of Diseases and Related Health Problems, Tenth Edition [ICD-10] codes I10, I11, I12, I13, I14, and I15), hyperlipidemia (ICD-10 code E78), diabetes mellitus (ICD-10 codes E10, E11, E12, E13, and E14), and chronic kidney disease (CKD) (ICD-10 code N18), from electronic medical data at our institution.

### Data availability

The datasets generated during and/or analyzed during the current study are available from the corresponding author on reasonable request.

### Statistical analysis

Categorical variables are presented as numbers and percentages, and were compared using the chi-square test or Fisher exact test. Continuous variables are expressed as a mean ± standard deviation or median (interquartile range). Based on their distributions, continuous variables were compared using the Student t-test or Wilcoxon rank-sum test. To analyze factors associated with AF, we used a multivariable logistic regression model (model 1) involving the following potential independent, clinically relevant variables: age >70 years; sex; LV geometric remodelings that was defined as ordered variables (normal geometry, concentric remodeling, concentric hypertrophy, and eccentric hypertrophy); and the presence of comorbidities such as ishemic heart disease, hypertension, diabetes mellitus, hyperlipidemia, CKD and overweight (body mass index >25 kg/m^2^). The adjusted odds ratios and 95% confidence intervals were calculated. To show the association of two variables (LAD and LVMI, LAD and RWT, LVEF and LVMI, and LVEF and RWT), we presented scatter plots, lines of best fit, and correlation coefficients. To visualize the effect size of each variable, the LogWorth (−log10(p-value)) scales were presented^18^. In LogWorth sclaes, a highly significant p-value had a large LogWorth value and a nonsignificant p-value had a low LogWorth value^18^. We generated the second multivariable regression model (model 2) including the LA size and LVEF to visualize the effect size of the LA size and LVEF as the LogWorth scales. In addition, we generated a multivariable logistic regression model using the same variables in AF for adjustment to analyze factors associated with a high LAVI and a low LVEF. Finally, from a logistic regression model with nominal responses (AF or not) using LVEF and LAVI, we generated a formula for linear combinations of the response levels (x = a + b x LVEF + c x LAVI) and prediction formulas for the response levels predicting the presence of AF (1/ (1 + Exp(x)) and the absence of AF (1/(1 + EXP(-x)), then we compared the two levels and predicted the presence or absence of AF according to the larger response level. We showed a receiver-operating curve with an area under the curve and provided the sensitivity and 1-specificity of this prediction formulas.

All statistical analyses were performed using JMP, version 13.2 (SAS Institute Inc., Chicago, IL, USA).

## Results

### Patients’ characteristics according to the LV geometric patterns

Baseline characteristics of patients are provided in Table 1. Among patients’ characteristics, patients with a normal geometry were significantly younger than who had abnormal geometry. The ratio of men were 52.4%, 58.3%, 48.3%, and 45.0% in patients with a normal geometry, concentric remodeling, concentric hypertrophy, and eccentric hypertrophy, respectively. Patients with concentric remodeling, concentric hypertrophy, and eccentric hypertrophy had high rates of hypertension, hyperlipidemia, diabetes mellitus, aortic valve stenosis, and CKD (Table 1).

**Table 1:** Baseline characteristics of the study subjects.

	Total (n = 4,014)	Normal geometry (n = 3,046)	Concentric remodeling (n = 437)	Concentric hypertrophy (n = 149)	Eccentric hypertrophy (n = 382)	p
Age, yr, SD	66.3, 15.9	64.5, 16.3	72.8, 12.1	71.9, 12.6	70.3, 13.6	<0.0001
Male, %	52.2	52.4	58.3	48.3	45.0	0.0014
Diabetes, %	29.9	27.3	40.3	45.6	33.0	<0.0001
Hypertension, %	57.0	50.6	74.4	89.3	76.7	<0.0001
Hyperlipidemia, %	29.0	26.6	36.6	42.3	35.1	<0.0001
Aortic stenosis, %	3.2	1.3	11.0	11.4	5.8	<0.0001
Aortic regurgitation, %	3.1	2.0	1.1	9.4	11.5	<0.0001
Mitral stenosis, %	0.2	0.2	0.2	0.7	0.5	0.340
Mitral regurgitation, %	3.6	2.5	1.8	4.7	13.0	<0.0001
Chronic kidney disease, %	14	10.5	20.1	38.9	25.9	<0.0001
Overweight, %	27.7	26.9	30.1	33.6	29.6	0.1419

Abbreviation: SD = standard deviation.

### Baseline characteristics of echocardiography

Baseline characteristics of echocardiographic findings are provided in Table 2. The mean LVEFs were 62.7, 62.6, 60.8, and 53.8% in patients with a normal geometry, concentric remodeling, concentric hypertrophy, and eccentric hypertrophy, respectively. The mean LAVIs were 22.5, 23.8, 33.3, and 37.0 mm/m^2^ in patients with a normal geometry, concentric remodeling, concentric hypertrophy, and eccentric hypertrophy, respectively.

**Table 2:** Baseline transthoracic echocardiography results.

	Total (n = 4,014)	Normal geometry (n = 3,046)	Concentric remodeling (n = 437)	Concentric hypertrophy (n = 149)	Eccentric hypertrophy (n = 382)	p
LVDd, mm	46.7, 6.1	46.4, 4.8	40.7, 4.1	46.2, 4.9	55.6, 7.6	<0.0001
E/e’	11.6, 4.8	10.8, 4.1	13.3, 5.4	17.3, 7.0	14.3, 5.7	<0.0001
LAD, mm	35.6, 7.0	34.7, 6.5	35.4, 6.8	40.0, 7.3	41.1, 7.7	<0.0001
LAVI, ml/m^2^	24.4, 14.8	22.5, 12.9	23.8, 12.5	33.3, 17.2	37.0, 21.6	<0.0001
LVEF, %	61.7, 7.6	62.7, 6.0	62.6, 4.9	60.8, 7.5	53.8, 14.5	<0.0001

Abbreviations: LVDd = left ventricular diastolic dimension; LAD = left atrial diameter; LAVI = left atrial volume index; LVEF = LV ejection fraction.

### Prevalence of AF

**Figure 2:**
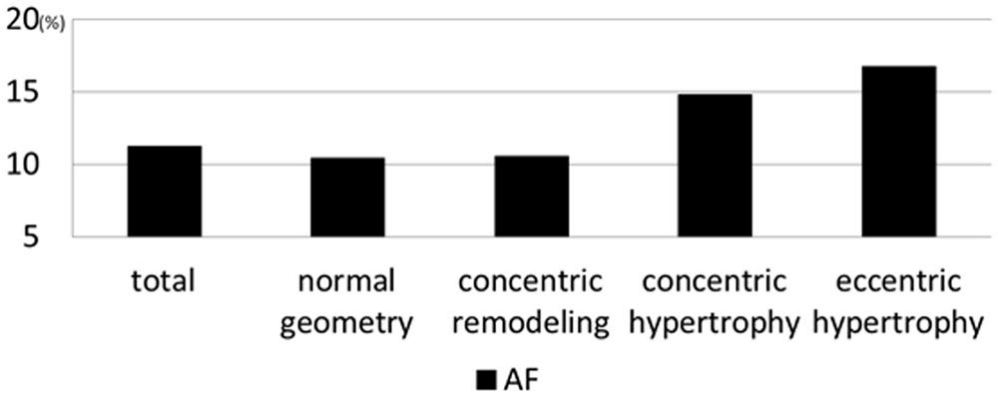
Prevalence of atrial fibrillation (AF) with each geometry patients.

Overall, 3460 patients had a normal sinus rhythm, 455 had AF, 95 had a pacemaker rhythm, and 15 had other rhythms. The prevalence rates of AF were 10.4, 10.5, 14.8, and 16.8% in patients with a normal geometry, concentric remodeling, concentric hypertrophy, and eccentric hypertrophy, respectively (p < 0.0001, Fig. 2).

### Association among LV geometry, LA size, and LVEF

**Figure 3:**
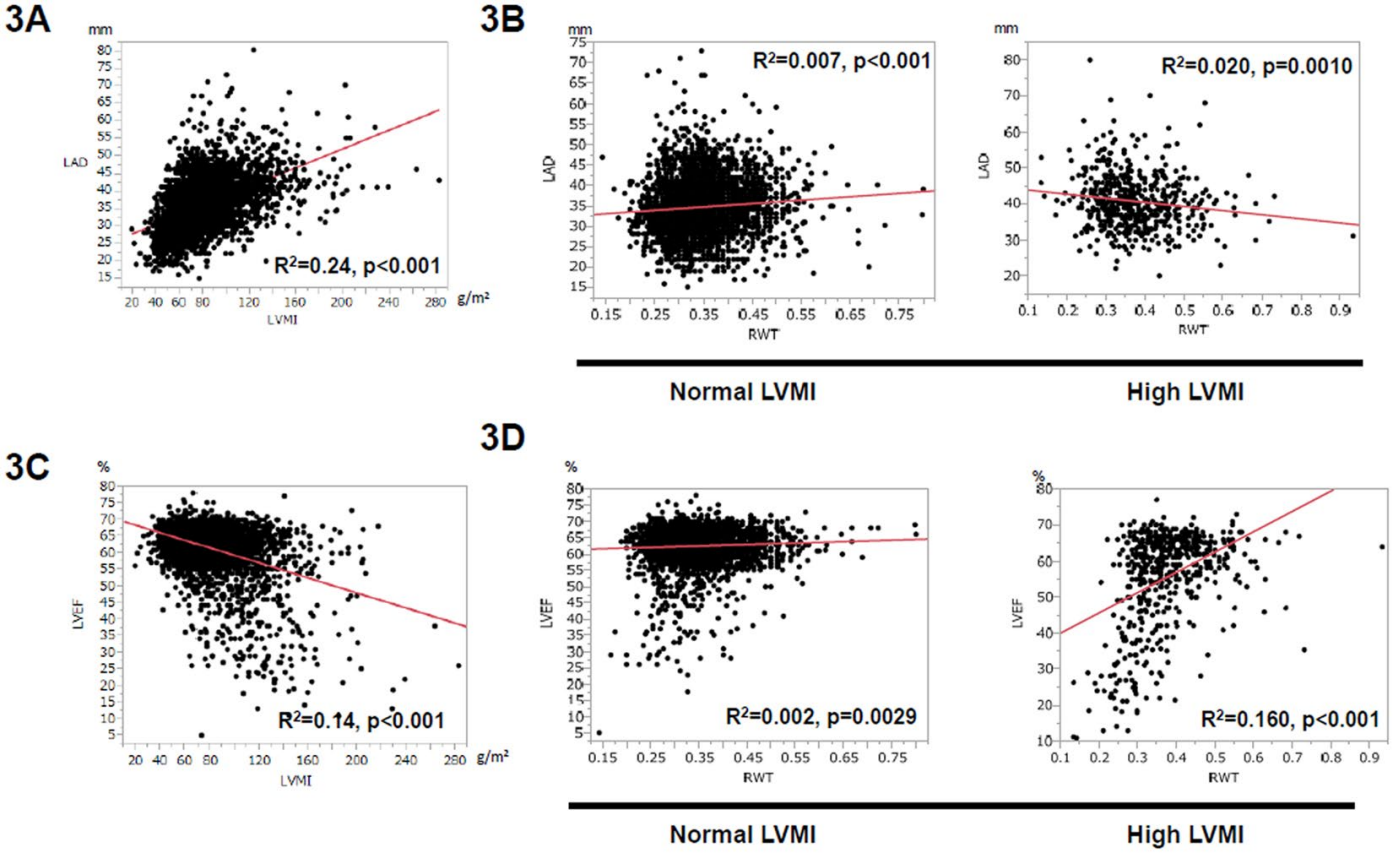
Association with LVMI, RWT, LA diameter, and LVEF. (**A**) Association between LVMI and LA diameter (LAD). Scatter plots show the positive correlation between LVMI and LAD. R^2^ = 0.24, p < 0.001. R = correlation coefficient. (**B**) Association between RWT and LAD. In patients with normal LVMI (left panel), scattered plots showed small correlation between RWT and LA diameter. R^2^ = 0.007, p < 0.001. In high LVMI (right panel), scatter plots showed small but, negative correlation between RWT and LA diameter. R^2^ = 0.020, p = 0.0010. (**C**) Association between LVMI and LVEF. Scatter plots showed the negative correlation between LVMI and LVEF. R^2^ = 0.14, p < 0.001. (**D**) Association between RWT and LVEF. In normal LVMI (left panel), scatter plots showed small correlation between RWT and LVEF. R^2^ = 0.002, p = 0.0029. In high LVMI (right panel), scatter plots showed small but, positive correlation between RWT and LVEF. R^2^ = 0.160, p < 0.001.

In order to investigate the underlying link between AF and LV geometry, we evaluated the relationship between LVMI, RWT, LVD, and LVEF. By using scatter plots, we visualized the association (Fig. 3). LAD was significantly related to the high LVMI (Fig. 3A). In contrast, LAD was small but positively related to high RWT when LVMI was normal (Fig. 3B), and negatively related to high RWT when LVMI was high (Fig. 3B). LVEF was significantly negatively correlated with LVMI (Fig. 3C). With regards to RWT, LVEF was only slightly correlated with RWT when LVMI was normal (Fig. 3D), but positively correlated with RWT when LVMI was high (Fig. 3D), showing that the relationship between LVEF and RWT was different for normal and high LVMI.

### Factors associated with AF and underlying LA enlargement and a low LVEF

**Figure 4:**
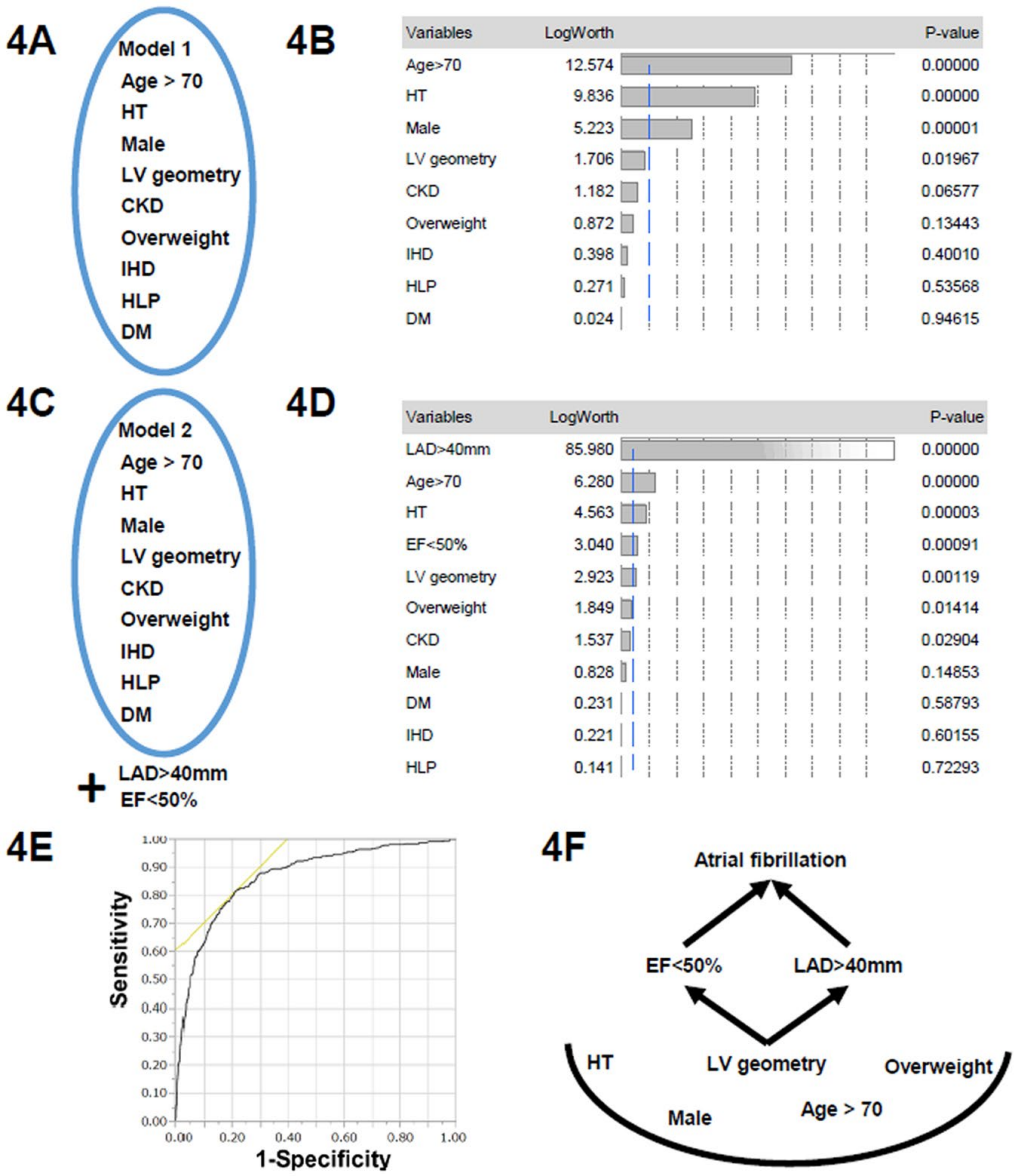
(**A**) Model 1 in Table 3 includes factors included age >70 years; sex; LV geometric remodelings that were defined as ordered variables (normal geometry, concentric remodeling, concentric hypertrophy, and eccentric hypertrophy); and the presence of comorbidities such as ischemic heart disease (IHD), hypertension (HT), diabetes mellitus (DM), hyperlipidemia (HLP), CKD and overweight (body mass index >25 kg/m^2^). (**B**) LogWorth scales of multivariate model 1 in Table 3. The p-values were transformed to the LogWorth (−log10(p-value)) scale. Hence, any LogWorth above 2 corresponds to a p-value below 0.01. A LogWorth of zero corresponds to a nonsignificant p-value of 1. (**C**) Model 2 includes factors included as factors for model 1, as well as left atrial enlargement [LAD (left atrial diameter) >40 mm] and low LVEF (EF <50%). (**D**) LogWorth scales of multivariate model 2. (**E**) Results of receiver operating characteristic (ROC) curve analysis. For predicting the presence of AF, the formula was generated from a linear regression (x = 3.55296 + 0.0208 × LVEF +− 0.09732 × LAVI). The prediction formulas for the response levels predicting the presence of AF and the absence of AF were (1/(1 + Exp(x)) and (1/(1 + EXP(−x)), respectively. Then we compared the two levels and predicted the presence or absence of AF according to the larger response level. The ROC curve shows an area under the curve of 0.871, and when x = 2.1433, the sensitivity is 0.8044 and 1-specificity is 0.6042. (**F**) A proposed schema for the relationship between LV geometry, LVEF, LA enlargement, and AF.

According to the results of the multivariate logistic regression analysis (model 1, Fig. 4A), the following variables were significantly associated with AF: age >70 years, male sex, hypertension, and LV geometric patterns (Table 3 and Fig. 4B). When we included two additional echocardiographic parameters, LA enlargement (LAD >40 mm) and a low LVEF (LVEF <50%), into a multivariate model for the presence of AF (model 2, Fig. 4C), LA enlargement was the echocardiographic parameter most associated with AF, and a low LVEF was the second most associated parameter (Fig. 4D), superior to the LV geometry. Finally, we sought the associated factors for LA enlargement and low LVEF. Table 4 shows the significant association with LV geometry and a high LAVI or LVEF. Finally, the presence of AF was well predicted by the values of LAVI and LVEF by receiver operating curve analysis (area under the curve 0.87; Fig. 4F). These indicated that the LV morphology was linked to the LV function and LA size (schematic in Fig. 4F).

**Table 3:** Factors associated with AF according to multivariate logistic regression analysis.

	AF		
	Multivariate OR	95% CI	P
Age >70 years	2.16	1.75–2.68	<0.0001
Male sex	1.67	1.35–2.06	<0.0001
HT	2.22	1.74–2.84	<0.0001
DM	1.00	0.80–1.24	0.987
CKD	0.77	0.57–1.02	0.078
HLP	0.94	0.75–1.18	0.615
Overweight	1.18	0.99–1.47	0.13
**LV geometry**			
Normal geometry	1 (Reference)		
Concentric remodeling	0.75	0.53–1.03	0.085
Concentric hypertrophy	1.10	0.66–1.75	0.683
Eccentric hypertrophy	1.45	1.06–1.96	0.018
Concentric remodeling	1 (Reference)		
Concentric hypertrophy	1.47	0.83–2.54	0.179
Eccentric hypertrophy	1.96	1.28–2.94	0.001
Concentric hypertrophy	1 (Reference)		
Eccentric hypertrophy	1.31	0.78–2.28	0.309

Abbreviations: OR = odds ratio; CI = confidence interval; RWT = relative wall thickness; LVMI = left ventricular mass index; HT = hypertension; DM = diabetes mellitus; CKD = chronic kidney disease, HLP = Hyperlipidemia.

**Table 4:** Factors associated with a high LAVI and a low LVEF according to multivariate logistic regression analysis.

	A high LAVI			A low LVEF		
	Multivariate OR	95% CI	P	Multivariate OR	95% CI	p
Age >70 years	3.29	2.43–4.47	<0.0001	0.85	0.63–1.14	0.288
Male sex	0.95	0.73–1.25	0.751	2.14	1.55–2.94	<0.0001
HT	2.14	1.53–2.99	<0.0001	4.60	2.93–7.23	<0.0001
DM	1.19	0.89–1.60	0.242	1.08	0.78–1.49	0.623
CKD	0.95	0.67–1.35	0.789	1.07	0.74–1.53	0.705
HLP	0.658	0.47–0.89	0.007	1.24	0.91–1.70	0.166
Overweight	0.91	0.68–1.24	0.574	0.72	0.51–0.99	0.045
**LV geometry**						
Normal geometry	1 (Reference)			1 (Reference)		
Concentric remodeling	1.03	0.66–1.61	0.872	0.41	0.19–0.86	0.018
Concentric hypertrophy	3.36	2.05–5.51	<0.0001	1.48	0.74–2.96	0.259
Eccentric hypertrophy	5.77	4.20–7.98	<0.0001	11.3	8.18–15.5	<0.0001
Concentric remodeling	1 (Reference)			1 (Reference)		
Concentric hypertrophy	3.24	1.77–5.94	0.0001	3.59	1.37–9.37	0.008
Eccentric hypertrophy	5.56	3.44–8.99	<0.0001	27.5	13.1–57.9	<0.001
Concentric hypertrophy	1 (Reference)			1 (Reference)		
Eccentric hypertrophy	1.71	1.02–2.87	0.040	7.66	3.83–15.3	<0.0001

Abbreviations: OR = odds ratio; CI = confidence interval; RWT = relative wall thickness; LVMI = left ventricular mass index; HT = hypertension; DM = diabetes mellitus; CKD = chronic kidney disease, HLP = Hyperlipidemia.

## Discussion

The main finding of the present study was that the prevalence of AF increased per the geometric remodeling patterns. The mechanism for why the prevalence of AF increased is unclear; however, cardiac remodeling manifests as changes in the cardiac size, shape, and function in response to aging, cardiac impairment, or an increased load^9^. Cardiac remodeling causes an increase in end-diastolic pressure and expansion of end-diastolic volume with diastolic and systolic dysfunction^9^. In the present study, patients with concentric remodeling, concentric hypertrophy, and eccentric hypertrophy were older than those with a normal geometry. In addition, patients with concentric remodeling, concentric hypertrophy, and eccentric hypertrophy had a higher E/e’. E/e’ is highest in patients with concentric hypertrophy^17^.

Concentric remodeling and hypertrophy were often caused by pressure overload that increased the RWT. This was an adaptation to normalize the systolic wall stress. However, LV was shown to be a substrate for LV diastolic dysfunction in concentric remodeling and an incremental risk for diastolic heart failure in concentric hypertrophy. Direct underlying triggers of AF was mainly due to atrial remodeling. Atrial remodeling is caused by multiple factors^19^. The left atrium is a thin-walled structure that connects the pulmonary veins and LV in diastole and pumps the LV (atrial kick) in end-diastole during sinus rhythm^15^. Previous work has highlighted the complex mechanisms contributing to AF progression^19–23^, as the morphological, electrical, or neurohormonal remodeling along with the ventricular responses were related. Diastolic impairment and an increasing diastolic filling pressure are related to the LA size. With an increase in LA filling pressures, the atrial wall stretches and enlarges. In this study, overweight was not significantly associated with AF, which was inconsistent with the previous studies^24^. One of the reasons may be the differences in baseline characteristics in Japanese population and in Western population. Body weights in Japan are much lower than those in the US and Europe. Other possible reason may be due to small numbers studied in our study, considering that a marginally significance existed.

Eccentric hypertrophy is typically induced by volume overload, such as in valvular heart disease or systolic heart failure. The prevalence of systolic dysfunction is mostly noted in eccentric hypertrophy. Systolic dysfunction affects LV geometry through enlargement of the LV chamber. A large LV and low LVEF cause congestion of the left atrium^21–23^. Consequently, LA enlargement in eccentric hypertrophy is suggested to be highest among groups with pressure and volume overload. A larger LA volume is associated with a higher risk of AF^15^. In addition, patients with AF and patients with a larger LA showed a higher LVMI and a low LVEF in a sub-study of the AFFIRM trial^25^. In our study analysis of both AF and non-AF populations, LA enlargement and LVEF were significantly related to LV geometric remodeling. Therefore, the rate of AF was increasing per the LV remodeling patterns. This was true for patients with non-valvular AF. The prevalence of LVH was high in patients with non-valvular AF^26^. Patel *et al*. reported that LAVI was associated only with LVMI, not RWT^21^. This is consistent with our present study which showed that the relationship between LA size and RWT was different for normal and high LVMI.

In addition, LAVI and LVEF accurately predicted the presence of AF in our study; therefore, we performed multivariate analyses excluding these co-linear factors. A linear association with LVMI and LA enlargement was observed; however, the effect of RWT on a low LVEF was different between patients with a normal LVMI and those with a high LVMI. Because LV geometric remodeling was clearly associated with LA enlargement and a low LVEF, a schematic in which that LV morphology was linked to the LV function and LA size is provided (Fig. 4F). Furthermore, the LV morphology was linked to the prognosis^7,26^. Thromboembolic events may be one of the mechanisms for the higher mortality in LVH^27^. Although there are reportedly many factors related to AF and LA enlargement, such as age, the presence of hypertension, and obesity^24^, LV geometric remodeling has an impact on the presence of AF through the association with LA enlargement and a low LVEF. However, atrial fibrillation can develop without left atrial enlargement^28^. Atrial size increases with time in patients with atrial fibrillation, without any structural or functional abnormality of LV and the valve^28^. Since our study is cross-sectional, we could not identify the cause-effect relationship in each patient. Longitudinal follow-up could address this issue in each patient.

In clinical practice, LVH is a risk factor in hypertensive patients^29^ and severe AS patients^30^. In our study, we provided the link between LV geometric patterns and AF prevalence through the contribution of atrial enlargement and decreased LVEF. AF and LA enlargement is a risk factor for ischemic stroke and may contribute to doubling all-cause^31^ and CV deaths^14^, and, in a specific condition, non-CV deaths^32^. However, anticoagulants were sub-optimally prescribed in these patients. Attention should be paid to paroxysmal AF in the LVH population to improve our daily practice.

There are several limitations to the present study. First, the subjects are heterogeneous because of the TTE and ECG findings. The ordering criteria for ECG and TTE were not set. Second, patients’ data were extracted from electronic medical data. A lack of information regarding exercise was an important limitation^24^. Third, we did not consider the effect of valvular diseases such as mitral valve regurgitation. Fourth, patients with short-lasting paroxysmal AF were excluded, because we included patients with AF when they had an AF rhythm according to an ECG examination. Lastly, the data presented provide only hypothesis-generating associations between LV geometry and AF. Since this study had a cross-sectional design, a cause-effect relationship could not be assessed. Further prospective studies are needed to determine the association of LA remodeling, LV geometric changes, LV function, mortality, and the time-course for the development of AF.

## Conclusions

The prevalence of AF was increasing according to LV geometric remodeling patterns in association with LA size and LVEF.
